# The non‐genetic paternal factors for congenital heart defects: A systematic review and meta‐analysis

**DOI:** 10.1002/clc.23194

**Published:** 2019-05-29

**Authors:** Jiayu Peng, Zhuo Meng, Shuang Zhou, Yue Zhou, Yujian Wu, Qingjie Wang, Jian Wang, Kun Sun

**Affiliations:** ^1^ Department of Pediatric Cardiology, Xinhua Hospital, School of Medicine Shanghai Jiao Tong University Shanghai China; ^2^ Department of Pediatric Cardiology, The Second Affiliated Hospital Yuying Children's Hospital of Wenzhou Medical University Wenzhou China

**Keywords:** congenital heart defects, meta‐analysis, paternal risk factors

## Abstract

**Background:**

Advances have been made in identifying genetic etiologies and maternal risk factors of congenital heart defects (CHDs), while few literatures are available regarding paternal risk factors for CHDs. Thus, we aim to conduct a meta‐analysis and systematic review about the non‐genetic paternal risk factors for CHDs.

**Methods:**

We searched the PubMed, MEDLINE, and Cochrane Library online databases and identified 31 studies published between 1990 and 2018 according to the inclusion criteria. Paternal risk factors were divided into subgroups, and summarized odd ratios (OR) were calculated.

**Results:**

Paternal age between 24 and 29 years decreased the risk of CHDs in the offspring (OR = 0.90 [0.82, 0.98]), while paternal age *≥* 35 years old increased the risk of CHDs (35‐39 years old: OR = 1.14 [1.09, 1.19], and *≥* 40 years: OR = 1.27 [1.14, 1.42]). Paternal cigarette smoking increased the risk of CHDs in a dose‐dependent way. Paternal wine drinking (OR = 1.47 [1.05, 2.07]) and exposure to chemical agents or drugs (OR = 2.15 [1.53, 3.02]) also increased the risk of CHDs. Some specific paternal occupations were also associated with increased risk for CHDs or CHD subtypes including factory workers, janitors, painters, and plywood mill workers.

**Conclusions:**

This meta‐analysis and systematic review suggested that advanced paternal age, cigarette smoking, wine drinking, exposure to chemical agents or drugs and some specific occupations were associated with an increased risk of CHDs. More measures should be taken to reduce occupational and environment exposures. At the same time, fertility at certain age and establishment of healthy life habits are strongly recommended.

## 1 INTRODUCTION

Congenital heart defects (CHDs) are groups of congenital cardiovascular disorders or diseases that affect about 1% of live births worldwide,^1^ which were also the leading cause of infant deaths.^2^ Over the past decades, there have been advances in the understanding of the risk factors for CHDs, that both genetic and non‐genetic risk factors are associated with the prevalence of CHDs. In the past, most investigations focused on maternal and genetic factors, while paternal factors attracted less attention. However, evidences suggested that paternal age, cigarette smoking, wine drinking, and occupational/environment exposures might have associations with various birth defects including CHDs.^3^, ^4^, ^5^, ^6^, ^7^, ^8^, ^9^, ^10^, ^11^, ^12^, ^13^, ^14^, ^15^ Therefore, we aimed to provide a current review of paternal factors for CHDs.

## 2 MATERIALS AND METHODS

This report of systematic review and meta‐analysis followed the instructions of Preferred Reporting Items for Systematic Reviews and Meta‐Analyses (PRISMA).^16^

### 2.1 Search strategy

We searched the PubMed, MEDLINE, and Cochrane Library online databases. We used the selected search terms and the Medical Subject Headings (MeSH) that were related to “congenital heart defect,” “risk factor,” “exposure,” and “paternal”. In addition to these search terms, individual risk factors also were included in the search terms (eg, “age,” “smoking,” and “drinking”). Reference lists of articles were reviewed to get more potentially eligible articles.

### 2.2 Inclusion criteria and exclusion criteria

We selected articles that (a) were observational epidemiologic study (case‐control and cohort study), (b) examined the association between any paternal exposures (eg, paternal age, paternal smoking, paternal drinking, paternal occupation, and paternal exposure to chemical agents) and CHDs overall or any one of the CHD subtypes in infants, (c) were written either in English, Chinese, or French, and (d) reported ORs (ie, risk ratios [RR] or odds ratios [OR]) and associated 95% confidence intervals (CIs) or had raw data available.

The exclusion criteria were: articles that (a) did not examine the association between any paternal exposures and any CHD subtypes in infants, (b) did not reported ORs and associated 95% CIs or had no raw data available, and (c) we could get the full text.

In the case of multiple publications using the same database, we selected the study that contained the most comprehensive information (eg, longest study periods or most CHD subtypes analyzed).

### 2.3 Data extraction

The studies meeting the inclusion criteria were independently reviewed by two authors (JP, JW) to extract study characteristics (eg, authors, year of publication, geographic region, periods of data collection, study design, sample size, exposure data, exposure period around pregnancy) and measures of association (eg, OR, RR). Measures of association not available in the original article were calculated based on raw data. Discrepancies between the authors were resolved by discussion.

### 2.4 Statistical analysis

We tested for heterogeneity across studies using Cochran's Q‐test. If there was an evidence of heterogeneity (*P* < .1), we used a random‐effects model. Otherwise, we used a fixed‐effects analysis. The statistical analyses were performed with Review Manager Version 5.3 (Cochrane Collaboration, Baltimore, Maryland).

Subgroup analysis was performed based on the different paternal factor, and sensitivity analysis was conducted. Publication bias was evaluated visually by funnel plots.

## 3 RESULTS

### 3.1 Study selection

**Figure 1:**
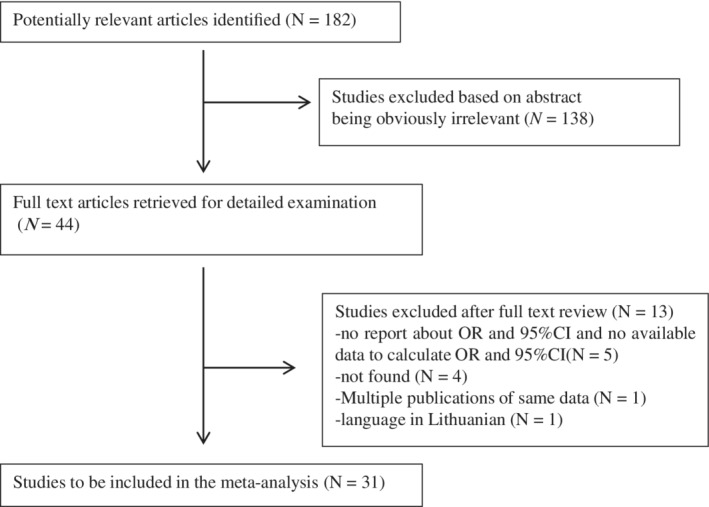
Flow chart of study selection process. CI, confidence intervals; OR, odd ratios

We identified 31 studies^3^, ^4^, ^5^, ^6^, ^7^, ^8^, ^9^, ^10^, ^11^, ^12^, ^13^, ^14^, ^15^, ^17^, ^18^, ^19^, ^20^, ^21^, ^22^, ^23^, ^24^, ^25^, ^26^, ^27^, ^28^, ^29^, ^30^, ^31^, ^32^, ^33^, ^34^ published between 1990 and 2018 according to the inclusion criteria. Study selection was summarized in Figure 1. Forty‐four studies were selected and retrieved for a full review. Five studies did not report OR and were excluded. Four studies were excluded since we had no access to full text. One study was only available in Lithuanian and was excluded. Finally, we included 31 studies for the meta‐analysis and systematic review.

### 3.2 Study characteristics

The characteristics of the included studies were summarized in Table 1. There were two cohort studies and 29 case‐control studies published between 1990 and 2018. The included studies had been performed in the United States, the United Kingdom, France, Egypt, Norway, the Netherlands, Sweden, Italy, Greek, Poland, Lithuania, Canada, China, and India. Risk factors were divided into five broad categories: paternal age, paternal cigarette smoking, paternal wine‐drinking, paternal occupation, and paternal exposure to chemical agents.

**Table 1:** The characteristics of the included studies

Study	Study period	Study location	Study design	No. of cases	No. of controls	Exposure	Cardiac defects	Exposure period	NOS
2018‐Liu	2004‐2014	China	Case‐control	4726	4726	Drinking	All CHDs	B3‐P3	7
2017‐Li	2013‐2014	China	Case‐control	119	239	Chronic disease	All CHDs	B6	6
						Exposure to occupational hazards			
2016‐Silver	1983‐2001	USA	Cohort	NA	NA	Age	VSD	B3‐P1	8
						Metal	VSD		
						Lead	VSD		
						Chlorinated hydrocarbons	VSD		
2016‐Ou	2004‐2013	China	Case‐control	4034	4034	Drinking	All CHDs	B3‐P3	7
				1566	4034	Drinking	VSD		7
				1028	4034	Drinking	ASD		
				212	4034	Drinking	PA		
				143	4034	Drinking	TGA		
2016‐Liu	2014‐2015	China	Case‐control	80	160	Drinking	All CHDs	B6	7
						Smoking			
						Age			
2016‐Abqari	2014–2015	India	Case‐control	400	754	Age	All CHDs	‐	6
2015‐Wang	2012‐2013	China	Case‐control	761	609	Age	All CHDs	B3‐P3	8
						Smoking			
						Drinking			
						Pesticides			
						Polychlorinated compounds			
						Phthalates			
						Alkylphenolic compounds			
						Bisphenol A			
						Heavy metals			
2015‐Qu	2004‐2012	China	Case‐control	3038	3038	Occupation	All CHDs	B3	8
2015‐Chen	2012–2013	China	Case‐control	435	574	Age	All CHDs	‐	6
2014‐Wijnands	2013‐	Netherlands	Case‐control	114	484	Phthalates	All CHDs	B1‐P2	6
2013‐Nie	2004‐2011	China	Case‐control	2568	2568	Antibiotics	All CHDs	B3	7
						Drinking			
						Chemical agent contact			
						Smoking			
						Virus infection			
						Age			
2013‐Fung	2008‐2011	Canada	Case‐control	1339	199	Smoking	All CHDs	B3‐P3	6
2013‐Deng	2010‐2012	China	Case‐control	284	422	Smoking	All CHDs	B3‐P3	6
				75	147	Smoking	Septal defects		
				72	147	Smoking	Conotruncal defects		
				31	147	Smoking	LVOTO		
				28	147	Smoking	RVOTO		
2012‐Snijder	2003‐2010	The Netherlands	Case‐control	421	477	Pesticides	All CHDs	B1‐P2	8
						Polychlorinated compounds			
						Phthalates			
						AlkylphenOlic compounds			
						Heavy metals			
2012‐Chehab	2006‐2010	France	Case‐control	2466	793	smoking	All CHDs	NA	6
2011‐Karatza	2006‐2009	Greek	Case‐control	157	208	Smoking	All CHDs	B1‐P3	7
2011‐Cresci	2008‐2010	Italy	Case‐control	330	330	Diagnostic X‐ray exposure	All CHDs	B3	6
						Drinking			
						Exposure to toxicants			
2010‐Kuciene	1995‐2005	Lithuania	Case‐control	261	1122	Smoking	All CHDs	‐	6
2010‐Green	1997‐2004	USA	Case‐control	740	5839	Age	PVS	‐	6
				1011	5839	Age	RVOTO		
2009‐Materna	1998‐2002	Poland	Case‐control	2451	6231	Age	All CHDs	‐	6
2007‐yang	1999‐2000	USA	Cohort	6797	5 360 532	Age	All CHDs	‐	7
2006‐Kuehl	1981‐1989	USA	Case‐control	142	3572	Age	HLHS	B6	6
2004‐Kazaura	1967‐1998	Norway	Case‐control	3628	13 668	Age	All CHDs	‐	6
2002‐Cedergren	1982‐1996	Sweden	Case‐control	269	524	Age	All CHDs	‐	7
2001‐Loffredo	1981–1989	USA	Case‐control	641	3549	Painting	Isolated membranous VSD	B3‐P3	6
2000‐Bassili	1995‐1997	Egypt	Case‐control	894	894	Age	All CHDs	‐	7
1997‐Ewing	1981–1989	USA	Case‐control	643	3551	Age	Isolated membranous VSD	B6	7
						Marijuana use	Isolated membranous VSD		
						Cocaine use	Isolated membranous VSD		
						Smoking	Isolated membranous VSD		
						Drinking	Isolated membranous VSD		
1996‐Aronson	1979‐1986	Canada	Nested Case–control cohort	9340	9340	Fire fighter	All CHDs	‐	8
1994‐Olshan	1952‐1973	UK	Case‐control	4110	8220	Age	VSD	‐	7
						Age	ASD		
1991‐Zhan	1986‐1987	China	Case–control	497	6222	Age	All CHDs	‐	7
1991‐Olshan	1952–1973	UK	Case‐control	1081	2272	Occupation	ASD	‐	7
				657	1213	Occupation	VSD		8
				1125	2309	Occupation	PDA		
				594	1256	Occupation	Other CHDs		

Abbreviations: ASD, atrial septal defect; HLHS, hypoplastic left heart syndrome; LVOTO, left ventricular outflow tract obstructions; NOS, Newcastle‐Ottawa Scale; PA, pulmonary atresia; PDA, patent ductus arteriosus; PVS, pulmonary valve stenosis; RVOTO, right ventricular outflow tract obstructions; TGA, transposition of great artery; VSD, ventricular septal defect.

B#, month before conception, B, unspecified time before conception, P#, month during pregnancy.

### 3.3 Paternal age

Eleven studies focused on paternal age as a risk factor for CHDs in offspring.^4^, ^5^, ^10^, ^11^, ^12^, ^13^, ^15^, ^19^, ^25^, ^30^, ^32^ Four studies evaluated the effect of advanced paternal age on the risk of CHDs, and the pooled OR is 1.02 (1.00, 1.04).

In addition, eight studies categorized paternal age into different age groups and we summarized the same age group, namely, <20, 20 to 24, 25 to 29, 30 to 34, 35 to 39, and ≥40 years of age. As shown in Table 2 and TABLE S1, paternal age older than 35 years was associated with higher risk of CHDs in offspring (OR for 35‐39 years: 1.14 [1.09, 1.19], OR for ≥40 years: 1.27 [1.14, 1.42]). On the contrary, paternal age of 25 to 29 years was associated with the lowest risk (OR = 0.90 [0.82, 0.98]).

**Table 2:** The results of subgroup analysis of non‐genetic paternal factors on congenital heart defects

Exposure	No. of cases	No. of controls	Summary odds ratio (95% CI)	Heterogeneity *P*‐value		Funnel plot
Age (years)	7137	860 802	1.02 (1.00, 1.04)	.04	RE	Symmetric
<20	495	228 352	1.06 (0.72, 1.54)	.01	RE	Symmetric
20‐24	2978	1 120 362	0.90 (0.80, 1.02)	<.0001	RE	Symmetric
25‐29	5745	1 740 888	0.90 (0.82, 0.98)	<.0001	RE	Symmetric
30‐34	4816	1 635 132	0.99 (0.90, 1.08)	.0002	RE	Symmetric
35‐39	2816	987 206	1.14 (1.09, 1.19)	.45	FE	Symmetric
≥40	2032	523 839	1.27 (1.14, 1.42)	.0001	RE	Symmetric
Smoking (cigarette/day)	8709	14 456	1.42 (1.17, 1.74)	<.0001	RE	Asymmetric
1‐9	434	597	1.19 (0.82, 1.71)	.003	RE	Asymmetric
10‐19	467	495	1.41 (1.20, 1.67)	.15	FE	Symmetric
20‐	1131	730	1.75 (1.10, 2.80)	<.0001	RE	Asymmetric
Drinking	13 406	16 430	1.47 (1.05, 2.07)	<.0001	RE	Symmetric
Toxicant	NA	NA	2.15 (1.53, 3.02)	<.0001	RE	Symmetric

Abbreviations: CI, confidence interval; FE, fixed effects model; NA, not available; OR, odds ratio; RE, random effects model.

### 3.4 Paternal cigarette smoking

Maternal‐smoking is now a well‐proved risk factor for CHDs.^35^ Similarly, paternal smoking also attracted growing concerns. Ten studies^7^, ^8^, ^9^, ^11^, ^14^, ^15^, ^20^, ^21^, ^23^, ^27^ evaluated the role of paternal smoking in the origin of CHDs and the summarized OR was 1.42 (1.17, 1.74) (Table 2, TABLE S2). Furthermore, based on the amount of cigarette smoking per day, the paternal smokers were also divided into three groups as follows: light smoking (1‐9 cigarettes per day), medium smoking (10‐19 cigarettes per day), and heavy smoking (≥20 cigarettes per day), and the pooled OR was 1.19 (0.82, 1.71), 1.41 (1.20, 1.67), and 1.75 (1.10, 2.80), respectively. This suggested that paternal smoking was associated with increased risk of having offspring with CHDs and this association was dose‐dependent.

### 3.5 Paternal wine drinking

Seven studies^6^, ^11^, ^14^, ^15^, ^17^, ^20^, ^21^ evaluated the effect of paternal alcohol consumption on CHDs. The summarized OR was 1.47 (1.05, 2.07) (Table 2, TABLE S3), indicating that paternal alcohol intake was a risk factor for CHDs in the offspring. However, the definition of paternal wine drinking was various from studies. The most common definition was defined by drinking capacity, that is, wine drinking mean a reported alcohol intake of on average at least 50 mL per day or per time without specifying wine.^6^, ^11^, ^15^, ^20^, ^21^ Others defined wine drinking by the amount of wine categories.^11^, ^14^ Only one study did not specify the definition of wine drinking.^17^

### 3.6 Paternal exposure to chemical agents or drugs

Seven studies^3^, ^11^, ^14^, ^15^, ^20^, ^22^, ^23^ evaluated the effect of paternal exposure to chemical agents or drugs on CHDs. These toxic chemical agents including pesticides, polychlorinated compounds, phthalates, alkyl phenolic compounds, bisphenol A, heavy metals,^15^ hydrocarbons,^3^ marijuana, and cocaine.^11^ After meta‐analysis, we found that paternal exposure to chemical agents or drugs had a strong association with increased risk of CHDs (OR = 2.15 [1.53, 3.02]) (Table 2, TABLE S4).

### 3.7 Paternal occupation

Some occupations like factory workers (left‐to‐right shunt CHDs [OR = 1.46, 95% CI: 1.23‐1.73] and left ventricular outflow tract obstruction CHDs [OR = 6.01, 95% CI: 1.05‐34.59], janitors ventricular septal defects [OR = 2.45], other heart defects [OR = 2.35], atrial septal defects [OR = 2.03]), painters (patent ductus arteriosus [OR = 2.34]) and plywood mill workers (patent ductus arteriosus [OR = 2.52]) might increase the risk of CHDs.^20^, ^34^ However, inconsistent results were shown in the investigations about the association between fire fighters and the risk of CHDs in offspring. An exploratory case‐control study from British Columbia reported statistically significant increased risk for ventricular and atrial septal defects among offspring of male fire fighters, compared to all other paternal occupations and to policemen.^36^ However, another investigation conducted in Metropolitan Toronto did not support the hypothesis of elevated risk of CHDs among the offspring of fire fighters.^33^

### 3.8 Other paternal risk factors

Apart from the above paternal risk factors, there are also several studies concerned about other paternal risk factors, such as chronic disease, viral infection, etc. Paternal chronic disease was another risk factor for CHDs (OR = 4.87, 95% CI: 1.23‐19.24), according to the findings of Li's investigation.^18^ And paternal virus infection (OR = 2.46, 95% CI: 1.13‐5.35), antibiotics usage (OR = l0.04, 95% CI: 1.28‐78.45) may also increase the risk of CHDs.^23^ On the other hand, evidences suggested that some paternal factors might not be the risk factors for CHDs. Paternal diagnostic X‐ray exposure may not increase the risk of CHDs (OR = 1.3, 95% CI: 0.8‐2.1).^14^

## 4 DISCUSSION

More and more evidence showed that not only maternal factors but also some paternal factors were associated with increased risk of CHDs. Nevertheless, there was little review or meta‐analysis focused on the non‐genetic paternal factors for CHDs, and our study made up this blank. We analyzed almost all the current literature and made a relatively comprehensive summary about the non‐genetic paternal factors for CHDs. After subgroup analysis, we found that advanced paternal age, cigarette smoking, wine drinking, some occupations, and exposure to chemical agents and drugs were still associated with the increased risk of CHDs.

Advanced paternal age was previously found to be associated with increased DNA mutations and chromosomal aberrations in sperm.^37^ Genetic changes in sperm associated with advanced paternal age could lead to an increased risk for birth defects in offspring.^10^ Consistent with these findings, we found that advanced paternal age (≥35 years) was associated with increased risk of CHDs. On the contrary, paternal age between 25 to 29 years decreased the risk of CHDs. This suggested that a certain reproductive age might be helpful to reduce the prevalence of CHDs, which could help to provide evidence for governmental health policy. In addition, these conclusions still need further cohort studies with larger sample to confirm.

Cigarette smoking is a well‐known teratogenic risk factor for birth defects and it can affect a number of developing structures.^35^ Nicotine, the main toxic agent during smoking, could affect sperm activity greatly and lead to chromosome aberration, which might affect the fetal development, and result in the occurrence of cardiac malformations.^38^ Besides, paternal smoking could induce maternal passive smoking, which could also increase the risk of CHDs.^39^ Consistently, Deng et al found that the avoidance behavior of paternal smokers might decrease the risk of selected CHDs.^7^

Apart from smoking, paternal wine drinking was also associated with increased risk of CHDs. However, drinking might be a temporary risk factor because Liu et al showed no evidence that wine‐drinking history would increase the risk of CHDs (OR = 1.087, 0.618‐1.913).^21^ The association between paternal wine drinking and CHDs in the offspring might need further validation in large cohort studies.

Some occupations like factory workers, painters, and plywood mill workers, probably suffered occupational exposures and Cresci's investigation suggested that occupational/environmental exposures increased the risk of CHDs.^14^ Several studies have shown that toxicant compounds could induce oxidative DNA damage, mutations, and chromosomal aberrations, such as DNA strand breaks and aneuploidy in human seminal fluid. And they have detected teratogenic, carcinogenic, and endocrine disrupting agents, such as pesticide residues, heavy metal organic solvents (benzene, toluene, and xylene), nicotine, aromatic hydrocarbons, and precursors of mutagenic nitrosamines in human seminal fluid.^40^ However, with the changing of natural and work environment, the situation may be different when it comes to how the current paternal occupation and exposure to chemical agents affect the prevalence of CHDs. And this needs further researches to explore.

The expose period defined by most identified studies is 3 months before conception.^3^, ^6^, ^7^, ^14^, ^15^, ^17^, ^20^, ^23^, ^24^, ^31^ The duration of spermatogenesis in human is 72 to 74 days, involving differentiation of the germ cells through several stages of meiosis and mitosis, some of which may be more vulnerable to cytotoxic damage or alterations in the DNA sequence.^28^ Thus, 3 months before conception could be a critical period of paternal risk factors for CHDs. However, the situation is different when it comes to smoking. In Liu's study, both paternal smoking history (OR = 2.687 [1.538‐4.692]) and paternal smoking half a year before pregnancy (OR = 2.889 [1.589‐5.254]) increased the risk of CHDs.^21^ Therefore, some paternal factors may have long‐term effects on CHDs.

This study identified articles mostly from the main continents, which were representative. However, there was still evidence of heterogeneity across studies even though subgroup analyses were performed. The probable reason might be the distinct subtypes of CHDs, which could obscure findings when subtypes were “lumped” into a common phenotype to increase study power. For publication bias, the funnel plot of smoking subgroup showed asymmetry, which indicated publication bias. However, for the rest of subgroups, the funnel plots were basically symmetric.

## 5 CONCLUSIONS

In conclusion, we summarized all the articles about non‐genetic paternal risk factors for CHDs and found that advanced paternal age, cigarette smoking, wine drinking, some occupations, and exposure to chemical agents and drugs would increase the risk of CHDs. It is important and urgent to encourage fertility at certain age, building a healthy life habit, beginning with quitting smoking and drinking, and trying to avoid occupational and environment exposures.

## CONFLICT OF INTEREST

The authors declare no potential conflict of interests.

## Supporting information


**TABLE S1** Forest plot: the association between paternal age and the prevalence of CHDs in offspring. CI: confidence intervals

**TABLE S2** Forest plot: the association between paternal cigarette smoking and the prevalence of CHDs in offspring. CI: confidence intervals

**TABLE S3** Forest plot: the association between paternal wine drinking and the prevalence of CHDs in offspring. CI: confidence intervals

**TABLE S4** Forest plot: the association between paternal exposure to chemical agents or drugs and the prevalence of CHDs in offspring. CI: confidence intervals
