# Supplementary figures and images for: The non‐genetic paternal factors for congenital heart defects: A systematic review and meta‐analysis
Source: Clin Cardiol. 2019 May 29;42(7):684–91. doi: 10.1002/clc.23194 (PMC6605632; doi:10.1002/clc.23194)

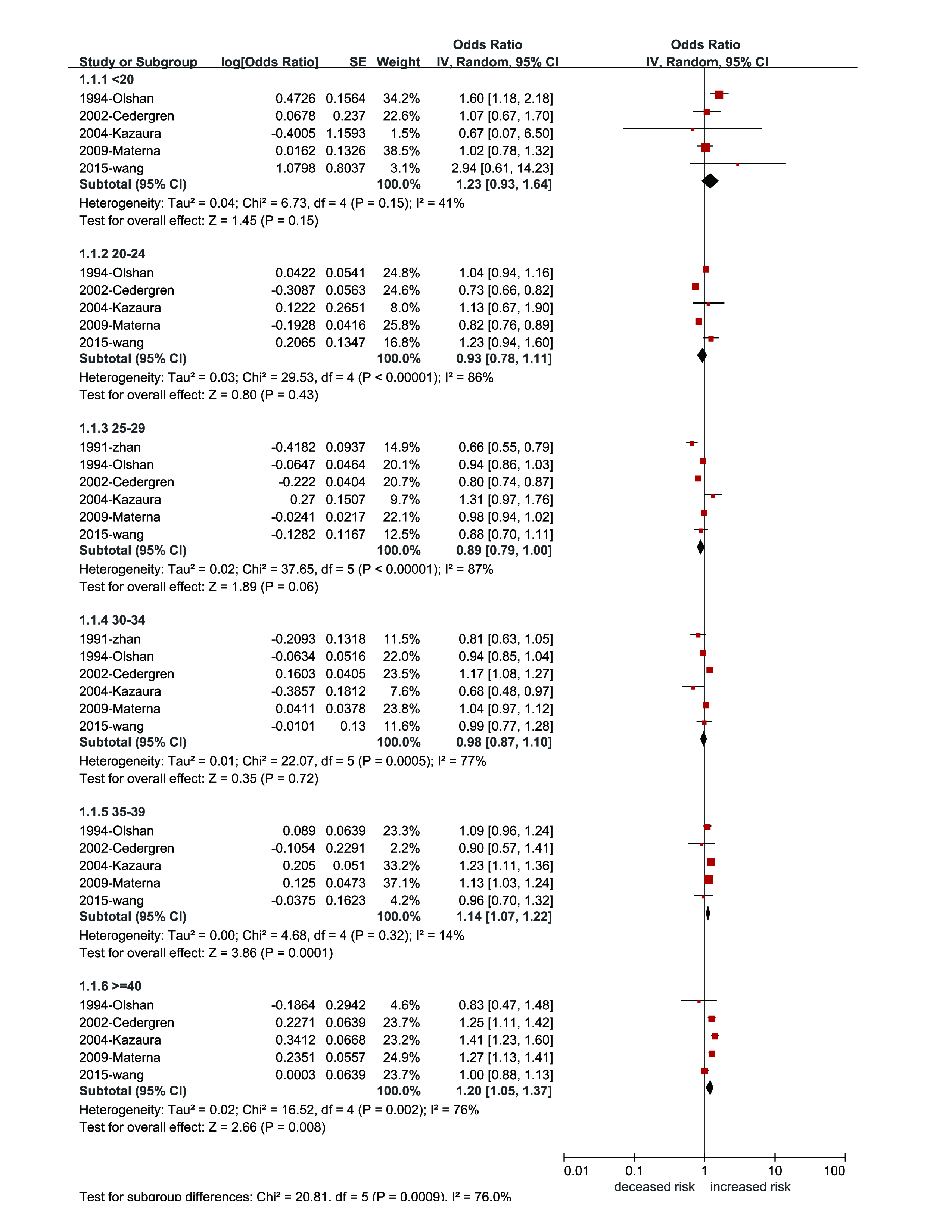

Supplement: Supplementary file 1 — TABLE S1 Forest plot: the association between paternal age and the prevalence of CHDs in offspring. CI: confidence intervals [file CLC-42-684-s001.tif]

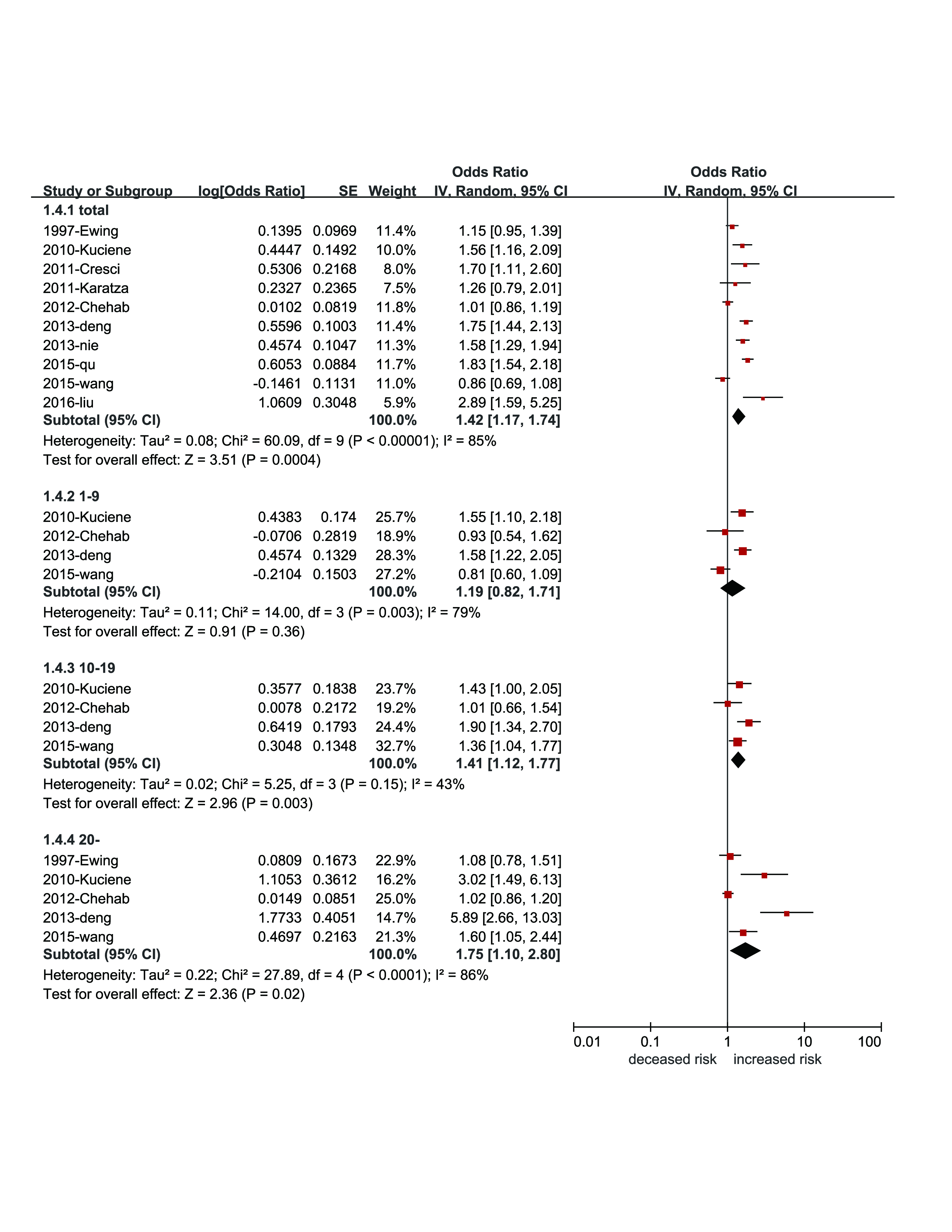

Supplement: Supplementary file 2 — TABLE S2 Forest plot: the association between paternal cigarette smoking and the prevalence of CHDs in offspring. CI: confidence intervals [file CLC-42-684-s002.tif]

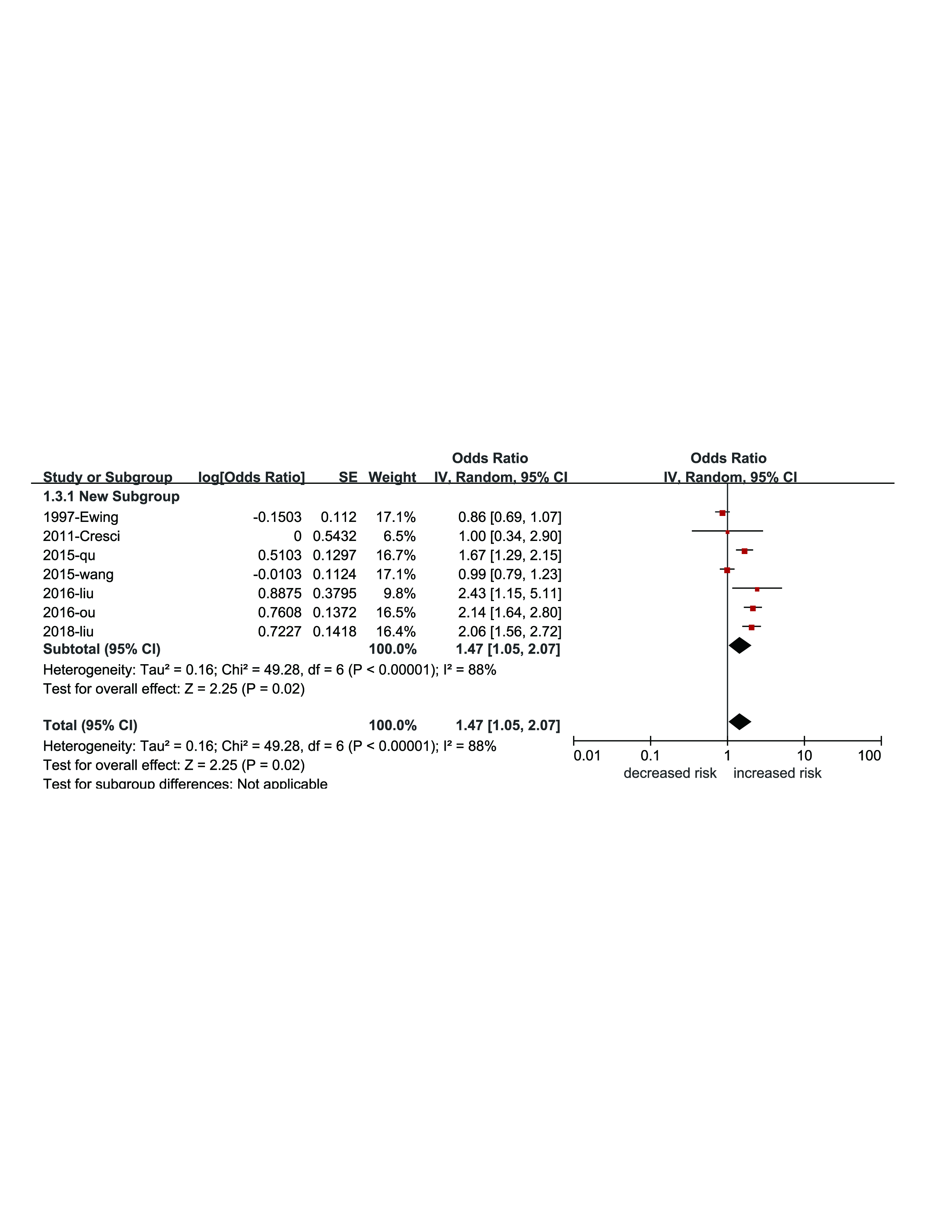

Supplement: Supplementary file 3 — TABLE S3 Forest plot: the association between paternal wine drinking and the prevalence of CHDs in offspring. CI: confidence intervals [file CLC-42-684-s003.tif]

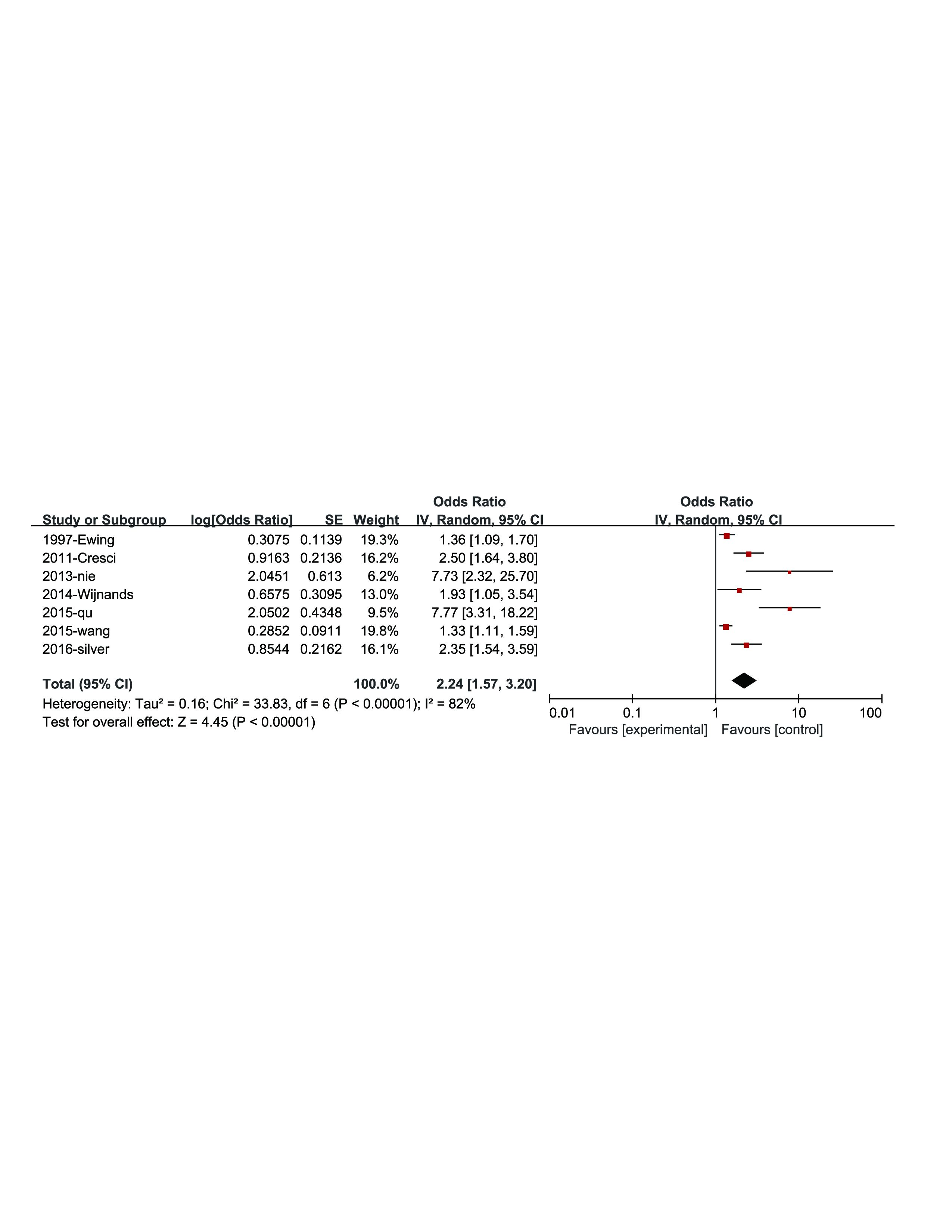

Supplement: Supplementary file 4 — TABLE S4 Forest plot: the association between paternal exposure to chemical agents or drugs and the prevalence of CHDs in offspring. CI: confidence intervals [file CLC-42-684-s004.tif]
